# Use of the Robust Design to Estimate Seasonal Abundance and Demographic Parameters of a Coastal Bottlenose Dolphin (*Tursiops aduncus*) Population

**DOI:** 10.1371/journal.pone.0076574

**Published:** 2013-10-09

**Authors:** Holly C. Smith, Ken Pollock, Kelly Waples, Stuart Bradley, Lars Bejder

**Affiliations:** 1 Murdoch University Cetacean Research Unit, Centre for Fish, Fisheries and Aquatic Ecosystems Research, School of Veterinary and Life Sciences, Murdoch University, Perth, Australia; 2 Marine Science Program, Nature Conservation Division, Department of Environment and Conservation, Perth, Australia; 3 North Carolina State University, Department of Biology, Raleigh, North Carolina, United States of America; University of St Andrews, United Kingdom

## Abstract

As delphinid populations become increasingly exposed to human activities we rely on our capacity to produce accurate abundance estimates upon which to base management decisions. This study applied mark–recapture methods following the Robust Design to estimate abundance, demographic parameters, and temporary emigration rates of an Indo-Pacific bottlenose dolphin (*Tursiops aduncus*) population off Bunbury, Western Australia. Boat-based photo-identification surveys were conducted year-round over three consecutive years along pre-determined transect lines to create a consistent sampling effort throughout the study period and area. The best fitting capture–recapture model showed a population with a seasonal Markovian temporary emigration with time varying survival and capture probabilities. Abundance estimates were seasonally dependent with consistently lower numbers obtained during winter and higher during summer and autumn across the three-year study period. Specifically, abundance estimates for all adults and juveniles (combined) varied from a low of 63 (95% CI 59 to 73) in winter of 2007 to a high of 139 (95% CI 134 to148) in autumn of 2009. Temporary emigration rates (γ') for animals absent in the previous period ranged from 0.34 to 0.97 (mean  =  0.54; ±SE 0.11) with a peak during spring. Temporary emigration rates for animals present during the previous period (γ'') were lower, ranging from 0.00 to 0.29, with a mean of 0.16 (± SE 0.04). This model yielded a mean apparent survival estimate for juveniles and adults (combined) of 0.95 (± SE 0.02) and a capture probability from 0.07 to 0.51 with a mean of 0.30 (± SE 0.04). This study demonstrates the importance of incorporating temporary emigration to accurately estimate abundance of coastal delphinids. Temporary emigration rates were high in this study, despite the large area surveyed, indicating the challenges of sampling highly mobile animals which range over large spatial areas.

## Introduction

Coastal bottlenose dolphins (*Tursiops spp*) are one of the most studied cetacean species mainly due to their widespread distribution and ease of accessibility. Much is known about their biology, social behaviour and population dynamics [1], [2], [3], [4], [5]. Various methods for estimating the abundance of dolphin populations have been developed and used routinely [1], [6], [7], [8], [9]. However, methods should be continuously refined based on the growing understanding of dolphin biology and behaviour to ensure that abundance estimates and population parameters are reliable and accurate [10], [11].

It is becoming clear that many coastal bottlenose dolphin populations include individuals with varying patterns of residency and ranging size. In some cases, individuals reside permanently in an area [12], while others transit through only occasionally [7], [9], [10]. In contrast, others temporarily emigrate leaving an area for a period of time (e.g. seasonally) but return more frequently than transients [13]. The varying degrees of individual residency have a large influence on abundance estimates at any given time and need to be incorporated into study designs and data analysis through statistical models.

Resident individuals may be particularly vulnerable to anthropogenic pressures such as habitat degradation, poor water quality, marine debris, anthropogenic noise, food provisioning from humans, competition with fisheries for prey and entanglement in fishing gear [14], [15], [16], [17]. Accurate assessments of abundance, distribution and life history parameters (such as survival and recruitment) are critical to understanding a population and its habitat use as well as identifying potential impacts of anthropogenic or natural pressures.

### Estimating abundance using capture-recapture techniques

For most delphinid species, particularly *Tursiops*, photographs of the dorsal fin are used for individual identification [18]. Fin shape and distinguishing marks, nicks and scars allow for their long-term identification, while markings on the surface of the fin and body are used for short-term identification [19]. Initial cataloguing of an identified individual is called the *capturing* process, and later resightings are defined as the *recapture* events. As identifying marks may change over time, it is necessary to record these changes and track modifications to avoid mis-identification [20].

While capture-recapture methods have remained the standard for identifying animals, there are a variety of sampling protocols for collecting the *captures*. These involve boat-based surveys conducted throughout zones of the study area while using varying temporal and spatial sampling designs. To take into account varying residency patterns and the opportunities available to capture all individuals within the population, careful thought must be given to the sampling regime, including sampling periods for and between *captures* and *recaptures*.

### Population models

**Figure 1:**
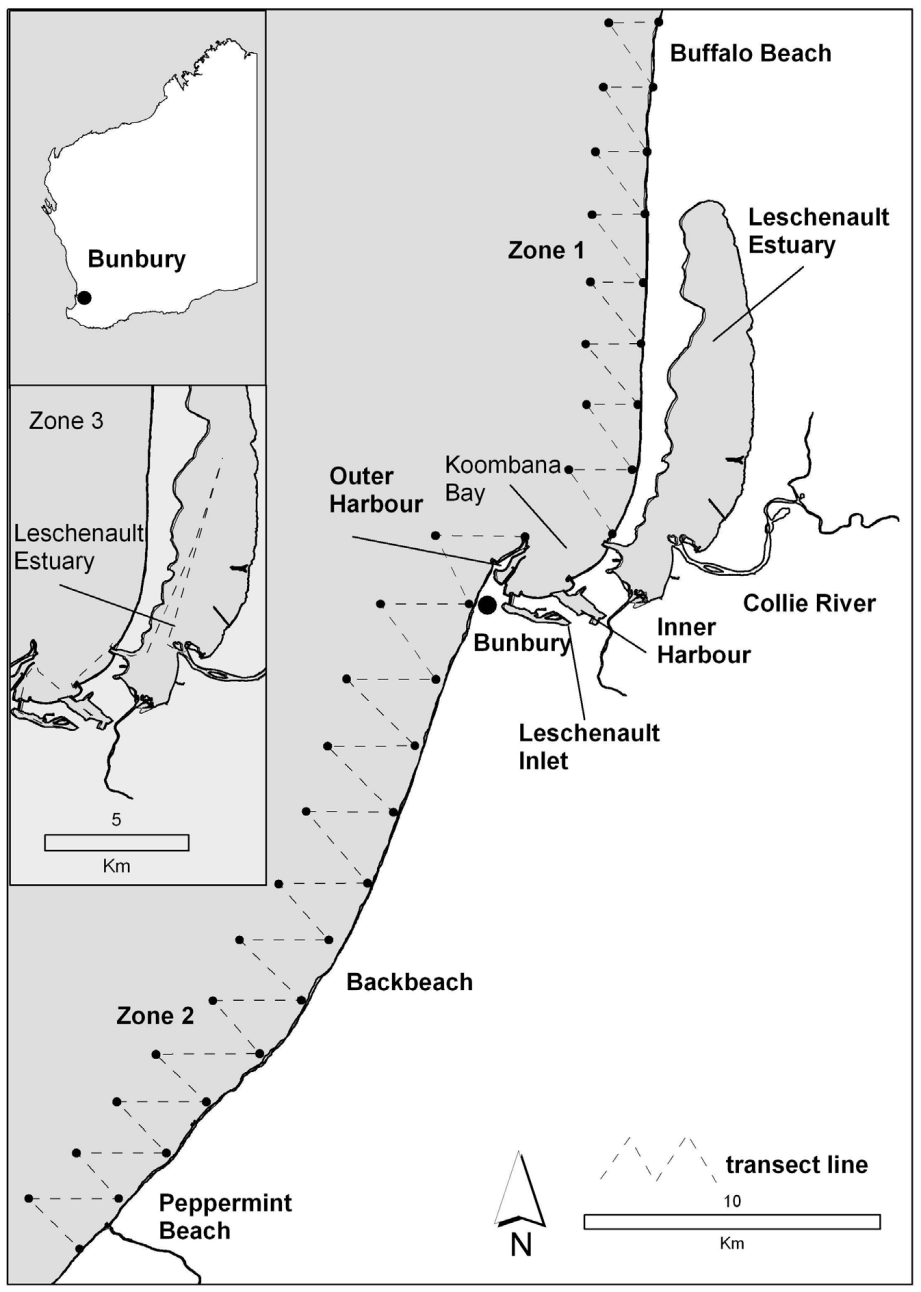
A map of the 120km2 study area in the near-shore environment around Bunbury, Western Australia. The extent of the three zones of transect lines (dashed zigzag line). Both Zone 1 (Buffalo Beach) and Zone 2 (Peppermint Beach) were coastal waterways, while Zone 3 (Bunbury Inner) consisted of inner waters including the Leschenault Inlet and Estuary, Inner and Outer Harbour, Koombana Bay and the lower reaches of the Collie River.

Statistical models used in capture-recapture studies are designed to calculate abundance estimates over multiple sampling periods [21]. Models are useful because they allow for population estimate calculations under complex parameterisations. Sampling designs must meet model assumptions, and the parameters must make sense biologically. Model parameters, e.g. survival and recruitment rates, should be customised as either constant or time-varying according to the characteristics and life history of the study population. A traditional definition of a population is “a group of organisms of the same species occupying a particular space at the same time” [22]. In this study, we considered the study population to include any bottlenose dolphins within our defined study area (Figure 1) at any time during the study.

**Closed versus open models.** There are two general types of models that have been used to describe bottlenose dolphin populations: closed and open. Closed population models are used when there are no population losses (through emigration or death) during the sampling period. To avoid violation of this assumption, closed models are best suited to studies where sampling can be conducted over a short period (e.g. a week). Open population models have been used more recently in cetacean studies [10], [23], [24]. They allow for permanent emigration, i.e. increases (births, immigration) and losses (mortality, emigration) to the population [21]. However, multiple movements in or out of the population, known as temporary emigration, is best accommodated in the Robust Design which combines characteristics of both open and closed population models [25], [26], [27]. Temporary emigration is defined as: (1) completely random; or (2) Markovian, where the probability of being unavailable for detection in primary period i depends on whether the animal was available (y") or unavailable (y’) for detection in primary period i – 1.Standard open models give rise to biased estimates if there is Markovian temporary emigration [21], [26].

The Robust Design incorporates open sampling events called “primary periods”, within which are a number of closed “secondary periods” [27]. Closure is assumed within primary periods but not between them. To fulfil this assumption, the Robust Design relies on secondary periods being close together, temporally [28]. Bottlenose dolphins have different sized home ranges (based on age and sex) that may result in unequal capture probabilities between individuals in the study area [29], [30]. Therefore, the capacity for the Robust Design to allow for temporary emigration is a very useful feature when estimating abundance for species that exhibit movement in and out of a study area.

The Robust Design has been used to estimate abundance and demographic parameters of other taxa, including multiple species of Plethodontid salamanders [31], Asian elephants (*Elephas maximus*) [32] and tigers (*Panthera tigris*) [33]. Similar to dolphins, these taxa may be available for capture in some sampling periods but not in others, as they move in and out of a study area. Likewise, the Robust Design has been used to estimate population abundance and parameters of several cetacean species, e.g. long-finned pilot whales *(Globicephala melas)* [34] Guiana dolphins (*Sotalia guianensis*) [35] and Irrawaddy dolphins *(Orcaella brevirostris)* [36]. This approach is particularly recommended for monitoring bottlenose dolphin populations because the model incorporates temporary emigration [10], [11], [13], [37], [38] which allows for the seasonal movements of this species [13], [39].

The aim of this study was to use a sampling design and population model that address the complexity posed by a coastal dolphin population comprised of both resident animals and temporary emigrants. In this study, we employed the Robust Design with two main objectives: (1) to examine seasonal abundances and demographic parameters of a coastal bottlenose dolphin population and (2) to explore the implications that residency and temporary emigration have on demographic parameter estimates.

## Materials and Methods

### Ethics statement

This study was carried out in strict accordance to the Murdoch University Animal Ethics Committee approval (W2009/06) and licensed as scientific research by the Department of Environment and Conservation (SF005811).

### Study area

The study was located next to the city of Bunbury, which is the fastest growing regional centre in Australia with a human population of 35,000 and is expected to increase to 100,000 by 2030 (Australian Bureau of Statistics 2010). Bunbury has one of the largest ports in Western Australia and plays a significant role in the State’s economy and trade (Department of Transport 2010). The port is the only export facility for local industry and produce from the surrounding rural areas. The study area covered 120 km^2^ of coastal waters around the city of Bunbury extending from Peppermint Beach to Buffalo Beach (Figure 1). The exposed coastal area was typified by open sandy beaches interspersed with limestone reef and an adjoining estuary. The inner-waters of Bunbury included the Leschenault Inlet, Leschenault Estuary, Bunbury Inner Harbour, Bunbury Outer Harbour, and the Collie River. The coastal study area covered 100 km^2^ and extended to approximately 1.5 km offshore, to a maximum water depth of 15 m, and with a linear distance of 50 km. The remaining area comprised inshore waters including the estuary, inlet, inner and outer harbours (20 km^2^). The benthic habitat in the study area was typical of temperate environments, including seagrasses, limestone reef, macroalgae communities and sand.

### Sampling methods

**Photo-identification capture-recapture technique.** Between March 2007 and November 2009, photo-identification was used during boat-based surveys as a capture-recapture method for estimating abundance, demographic parameters and movements of bottlenose dolphins. One field day of effort was defined as a *survey*, with each dolphin group encounter during a survey termed a *sighting*. Using a Nikon D 200 with Nikkor zoom lens 70–300 mm, photographs were taken of individual dolphins encountered along pre-determined transect lines (Figure 1). A sighting commenced when a dolphin or dolphin group were encountered along the transect line. A *zone* was defined as a pre-determined route that consisted of zig-zag transect lines that were followed during surveys. Surveys were conducted from a 5 m centre console research vessel driven at a speed of 8 to 12 kn with two to five observers (median =  4) present during each survey.

### Sampling design

**Line Transects.** Surveys were conducted year-round during three consecutive years and used repeated effort in three zones with pre-determined transects lines (Table 1; Figure 1). The transect lines within the three zones totalled 120 km with a strip width of approximately 250 m either side of the vessel. Zones 1 and 2 followed a zig-zag pattern to maximise coverage of the study area. However, this was not possible for Zone 3, where designated channels and shallow water governed the path of the transect lines. The aim of each field day was to complete all transect lines within one zone (Figure 1). Due to weather and time constraints, it was not possible to survey all three zones in a single day. A *sampling period* was defined as the time required to complete all three zones. A sampling period took a minimum of three days and a maximum of three weeks. This is equivalent to one *secondary sampling period* of the Robust Design. This study sampled year-round to account for possible seasonal temporary emigration, maximising the capture probability of all individuals in each sampling occasion and the likelihood of each individual being resighted annually.

**Table 1:** Sample sizes of the two-tiered data structure consisting of primary (seasons) and secondary sampling periods (no. of times all three zones of the study area were surveyed) of the Robust Design.

Year	Sampling Period	2007			2008				2009			
Seasons	Primary	Aut	Win	Spr	Sum	Aut	Win	Spr	Sum	Aut	Win	Spr
No. of times the study area was surveyed	Secondary	3	3	2	9	5	6	4	9	7	4	2

All surveys were conducted in Beaufort sea state ≤ 3. When a dolphin group was sighted, the research vessel would break away from the transect line to approach the dolphin(s) at which time the location (GPS coordinates) and dolphin group size were recorded. Sightings lasted for a minimum of five minutes (to allow determination of predominant activity) and until all the dorsal fins were photographed. Known individuals were photographed regardless of familiarity. Once all dolphins had been photographed, the vessel was repositioned on the transect line at the point where it was left and the search for other dolphin groups resumed.

### Data processing

Photographs were assessed according to their sharpness, contrast, angle and size of the dorsal fin in relation to the frame. Using these measures, only images of excellent and good quality were used for analyses [40], [41]. Images that could not reliably be used for individual identification were discarded. Individual dolphins were primarily identified based on the nicks and scars on the leading and trailing edges of the dorsal fin. Secondary features (such as pigmentation, natural fin shape, and scarring on the surface of the fin and peduncle) were also used for identification. The sampling regime allowed for the tracking of temporary/secondary markings (e.g. rake marks and/or lesions) that tended to fade after approximately six months.

### Characterisation of study subjects

**Age and sex.** The age of population members is an important consideration in abundance modelling, as it may influence the likelihood of individuals having distinguishable marks, capture probability and demographic and dispersal factors. In this study, the exact age of many individuals was unknown, therefore individuals were assigned to one of three mutually exclusive age classes (adults, juveniles and calves) according to body length. Calves were excluded from the analysis for two reasons: (1) because they lacked identifying marks and; (2) the natural mortality of calves is assumed to be high based on mortality of calves in the bottlenose dolphin population in Shark Bay, Western Australia (Mann *et al.* 2000). Juveniles were included in the analysis along with adults as they were sufficiently marked for recognition and recapture. Abundance estimates presented here refer to all juveniles and adults of both sexes combined.

Sex determination of adult female dolphins was usually based on a series of consistent sightings with a dependent calf. Confirming the sex of males and non-mother females was more challenging and relied on visual observation of the genital area or the genetic analysis from biopsy samples that were collected as part of a separate research project.

### Description of models

**Robust Design assumptions.** Many assumptions of the Robust Design are the same as for the standard open models (Williams et al. 2002), while also allowing for temporary emigration and with no requirement for population closure between primary periods. Specifically, the Robust Design allows a population to be open to immigration, emigration, births, and deaths between the primary periods, and assumes population closure over all the secondary periods within a primary period. Also, unlike standard models, the Robust Design allows for heterogeneity of capture probabilities because the secondary sampling periods occur close together.

The Robust Design allows for estimating: the probability of first capture ‘p’; the probability of recapture ‘c’; and the number of animals that are in the sampling area [N(i)]. For the intervals between primary sampling periods, the model also allows for parameter estimation of the: probability of apparent survival [S(i)], and two temporary emigration parameters which are the probability of emigration from the study area given that the animal was present in the last period [γ” (i)], and the probability of staying away from the study area given that the animal has left the survey area before this period [γ’(i)].

A Robust Design procedure within the software MARK [42] was chosen for analysis as it has the most flexibility in setting sampling occasions, parameters and incorporating unequal time intervals between sampling. Conventional methods of dolphin photo-identification use only permanent marks on the edge of the dorsal fin [18], [43]. In this study, all juveniles and adults were considered identifiable as each dorsal fin was distinct when the natural dorsal fin shape was used in conjunction with temporary marks on the surface of the dorsal fin and body, i.e. lesions and tooth rake marks [19]. Therefore, no adjustment was made for an unmarked proportion of the population.

**Robust Design structure.** Primary periods were based on the Australasian seasons: Summer (December-February), Autumn (March-May), Winter (June-August); and Spring (September-November). There were 11 primary periods (seasons) and 54 secondary periods in this three-year study (Table 1). The time it took to complete the secondary sampling periods varied and the intervals between secondary periods varied. Ideally, the time duration to complete a full secondary sampling period (i.e. all three zones within the entire study area) would have been shorter than we achieved (i.e., quicker (within days rather than weeks) but survey effort was constrained by weather. Again, we assumed that the population was closed within seasons (primary periods).

Data analysis was performed on the data available on juveniles and adults of both sexes. We used the Akaike Information Criterion (AIC) to evaluate model fit. The best fitting model was identified as having the lowest AICc [44].

## Results

### Survey effort and summary statistics

**Figure 2:**
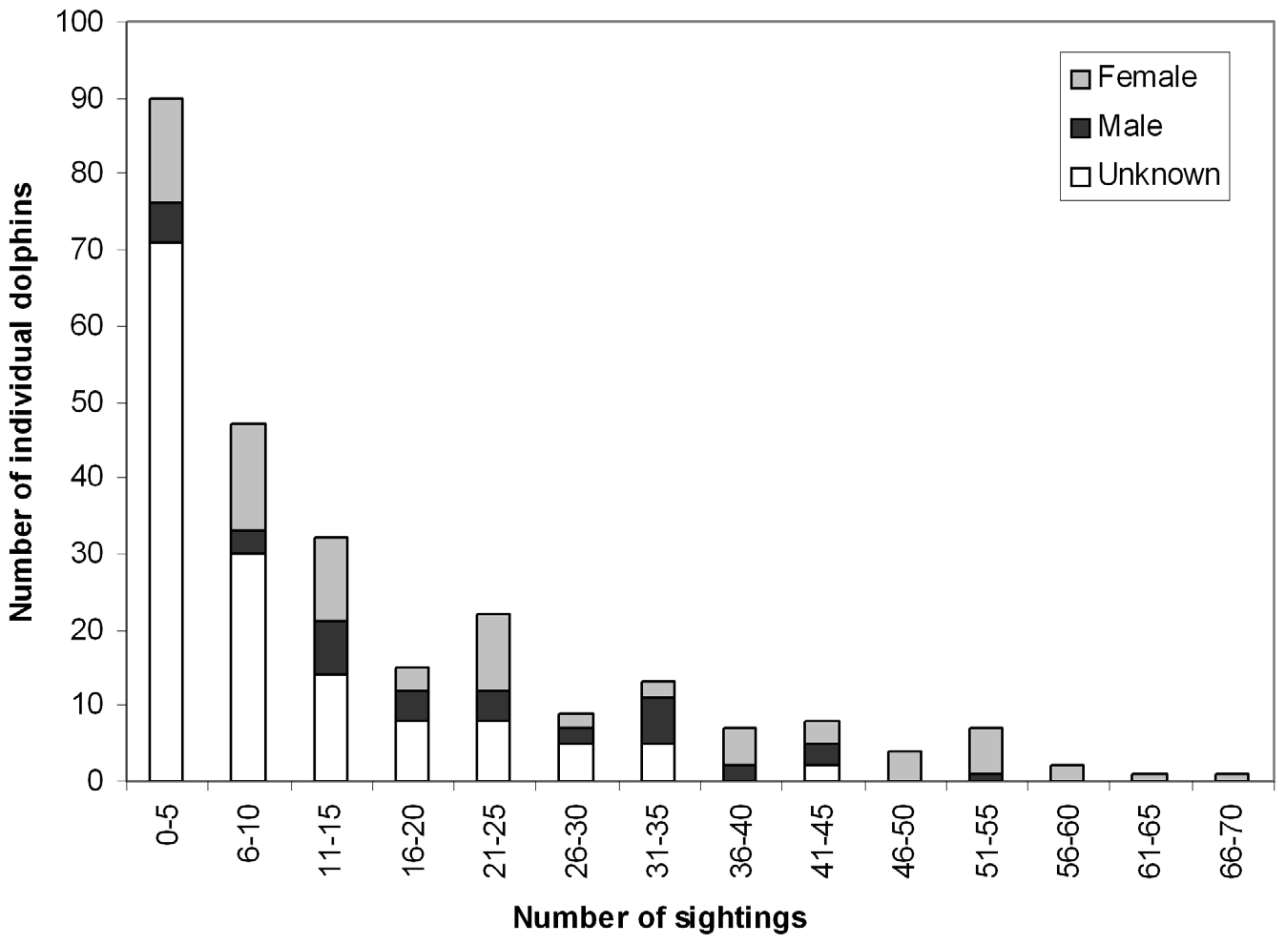
Sighting frequency of individually identified bottlenose dolphins (non-calves) March 2007-November 2009.

**Figure 3:**
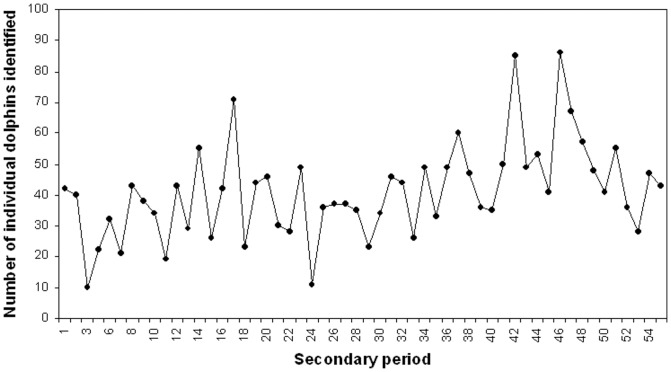
Number of individual dolphins (juveniles and adults) identified in each secondary period March 2007-November 2009.

A total of 201 surveys were completed over the three years, resulting in 544 dolphin group sightings (Tables 2 and 3). A total of 108, 219 and 357 dolphin groups were encountered along Zones 1, 2 and 3, respectively. In total, 172 individual dolphins were identified of which 39 and 133 were juveniles and adults, respectively (Table 4). The sighting frequency of individually identified dolphins ranged from 1 to 67 with a mean of 15 (± SE 1.3; Figure 2). The number of individual dolphins identified during each secondary sampling period ranged from 10–90 (mean  =  40; ±SE 2.0) (Figure 3).

**Table 2:** Summary of annual survey effort across months and number of zones surveyed between March 2007 and November 2009.

Year	No. of surveys	No. of months	No. of zones surveyed	No. of dolphin group sightings
2007	48	10 (Mar-Dec)	43	157
2008	100	12 (Jan-Dec)	85	137
2009	69	9 (Jan-Sept)	73	250

Dolphin group encounters are included as number of ‘sightings’ along transect lines.

**Table 3:** Total number of transect replicates per seasons for each zone.

	Summer	Autumn	Winter	Spring
	Dec-Jan-Feb	Mar-Apr-May	June-July-Aug	Sept-Oct-Nov
Zone 1	21	21	20	12
Zone 2	25	19	18	10
Zone 3	30	20	22	13

Zone 1: Buffalo Beach; Zone 2: Back Beach; and Zone 3: Bunbury Inner; Figure 1.

**Table 4:** Cumulative number of individually identified bottlenose dolphins during the study period (March 2007 and November 2009).

Year	Cumulative no. of juveniles identified	Cumulative no. of adults identified	Cumulative no. of dolphins identified
2007	22	57	79
2008	30	119	149
2009	39	133	172

Of the 172 individuals identified, 21 were documented as males (12%), 66 as females (39%) and 85 (49%) were of unknown sex. Forty one dolphins were sexed as females based on the presence of a dependent calf. Two male dolphins were confirmed through direct observation of the genitals. Biopsy and subsequent genetic analysis confirmed the sex of the additional 25 females and 19 males.

Adult females were sighted more often than adult males in all seasons (except during spring). Individual females were sighted, on average, on 7.33 (± SE 0.86) occasions while individual males were sighted, on average, on 4.55 (± SE 0.59) occasions in summer seasons. Spring was an exception where the reverse was observed: each adult male and female was, on average, observed on 3.80 (± SE 0.52) and 2.57 (± SE 0.31) occasions, respectively (Table 5).

**Table 5:** The mean number of sightings of individual adult male and adult female bottlenose dolphins for each Australasian season (March 2007 and November 2009).

	Autumn	Winter	Spring	Summer
Males (n = 21)	5.05 (±0.60)	2.30 (±0.61)	3.80 (±0.52)	4.55 (±0.59)
Females(n = 66)	5.03 (±0.49)	4.55 (±0.61)	2.57 (±0.31)	7.33 (±0.86)

Standard error is shown in brackets.

### Abundance, temporary emigration and survival estimates

**Figure 4:**
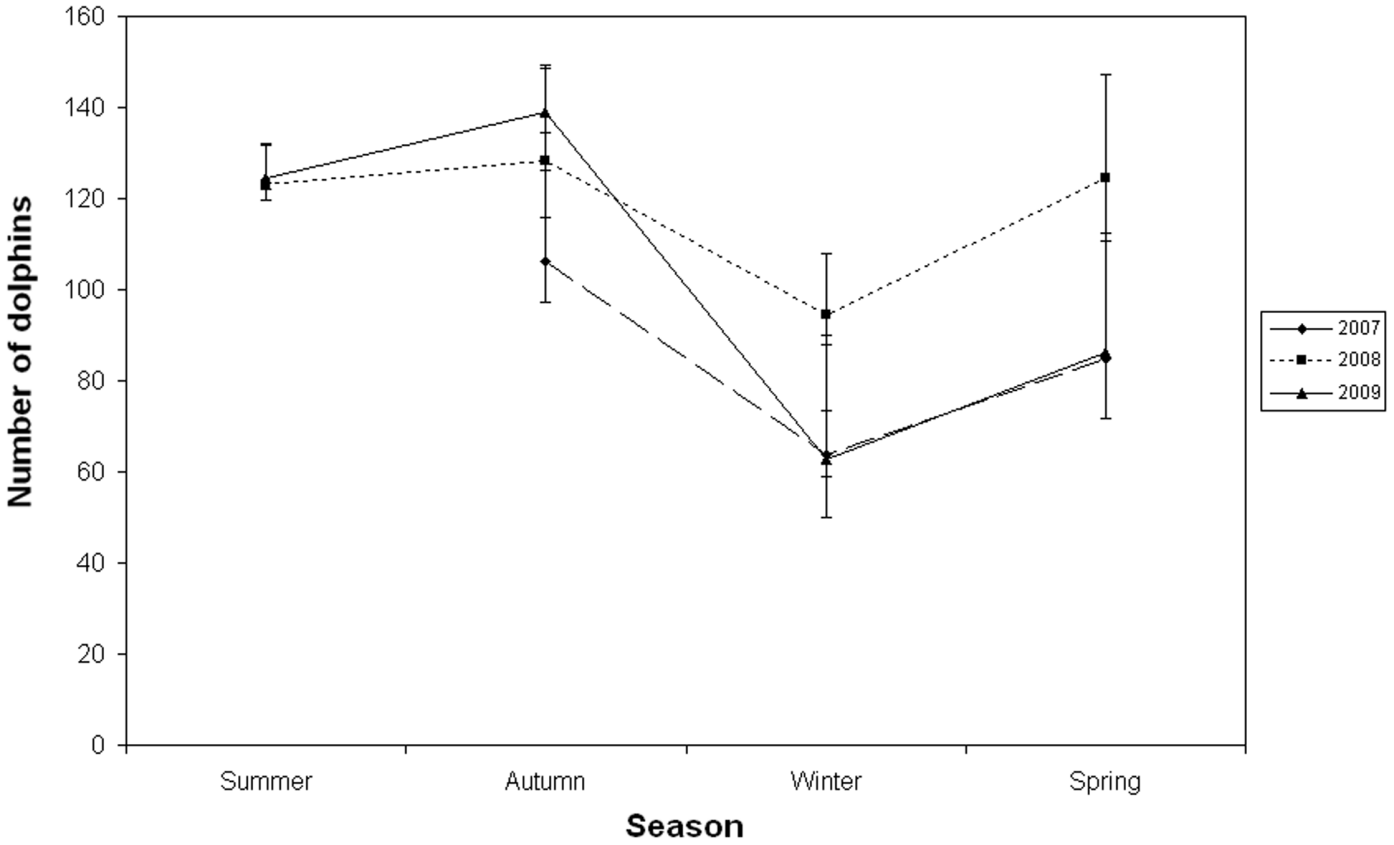
Seasonal abundance estimates of juvenile and adult (combined) bottlenose dolphins 2007-November 2009. 95% confidence intervals indicated by vertical bars.

The study population could not be considered geographically closed between seasons as some individuals were captured inconsistently across sampling seasons. Therefore, models that incorporated temporary emigration were included in the tested model sets. As assumed, models that did not incorporate a parameter for temporary emigration fit the data poorly (Table 6). Unfortunately, sex specific abundance estimates and demographic parameters could not be modelled separately because of the large proportion individuals that were of unknown sex. The best fitting model (φ_(t)_γ''_(s) ≠_γ'_(s)_ p_t_  = c_t_ N_t_), based on the lowest AICc, had apparent survival varying with time (rather than constant), seasonal Markovian temporary emigration (with time variation in emigration parameters γ'' and γ') and a different capture probability for each primary sampling occasion (Table 6). The temporary emigration rates of being absent based on the previous period state also being absent (γ') were high and ranged from 0.34 to 0.97 with a peak in spring and a mean value of 0.54±SE 0.11 (Table 7). The temporary emigration rates of being absent based on the previous period state being present (γ'') were lower, ranging 0.00–0.29, with a mean of 0.16±SE 0.04 (Table 7) and showed a strong seasonal effect with an estimated value of 0 during winter (Table 7) compared to 0.29 in autumn. Our model yielded a mean apparent survival estimate for juveniles and adults (combined) of 0.95±SE 0.02 and a capture probability of from 0.07 to 0.51 with a mean of 0.30 (± SE 0.04). Mean seasonal dolphin abundance estimates (juveniles and adults combined) varied according to season with consistently lower abundance estimated during winter and higher during summer and autumn across the three year study period (Figure 4). Specifically, abundance estimates ranged from a low of 63 (± SE 3.32; 95% CI 59 to 73) in winter 2007 to a high of 139 (± SE 3.39; 95% CI 134 to148) in autumn 2009 (Figure 4). This seasonal trend in abundance was apparent in all years (Figure 4).

**Table 6:** Capture-recapture models fitted to the capture histories of all juvenile and adult dolphins combined estimate parameters for population size (N), apparent survival (φ), temporary emigration (γ'', γ') and capture probability (p).

Models	Rank	AICc	δAICc	AICc weight	Model likelihood	Parameters	Deviance
**φ_ (t)_γ’’_(s) ≠_γ_(s)_ p_(t) = _c_(t)_**	**1**	**1297.9**	**0.0**	**0.908**	**1.000**	**83**	**5845.3**
φ_ (.)_γ’’_(s) ≠_γ’_(s)_ p_(t) = _c_(t)_	2	1302.5	4.6	0.092	0.102	74	5869.6
φ_ (.)_γ’’_(s) ≠_γ’_(.)_ p_(t) = _c_(t)_	3	1313.9	16.0	0.000	0.000	71	5887.6
φ_ (.)_γ’’_(t) ≠_γ’_(t)_ p_(t) = _c_(t)_	4	1325.0	27.1	0.000	0.000	83	5872.4
φ_ (.)_γ’’_(t) ≠_γ’_(.)_ p_(t) = _c_(t)_	5	1334.7	36.8	0.000	0.000	76	5897.5
φ_ (.)_γ’’_(t) = _γ’_(t)_ p_(t) = _c_(t)_	6	1356.6	58.7	0.000	0.000	75	5921.5
φ_ (.)_γ’’_(.) ≠_γ’_(t)_ p_(t)__ = _c_(t)_	7	1365.6	67.8	0.000	0.000	75	5930.6
φ_ (.)_γ’’_(.) ≠_γ’_(.)_ p_(t)__ = _c_(t)_	8	1373.5	75.6	0.000	0.000	68	5953.7
φ_ (.)_γ’’_(.) = _γ’_(.)_ p_(t)__ = _c_(t)_	9	1399.5	101.6	0.000	0.000	67	5981.8
φ_ (.)_γ’’_(t) ≠_γ’_(t)_ p_(.) = _ c_(.)_	10	1462.2	164.3	0.000	0.000	40	6102.0
φ_ (.)_γ’’0_ = _ γ’0 p_(t) = _ c_(t)_	11	1471.7	173.8	0.000	0.000	66	6056.2
φ_ (.)_γ’’_ (t) ≠_γ’_(.)_ p_(.) = _ c_(.)_	12	1472.1	174.2	0.000	0.000	33	6126.5
φ_ (.)_γ’’_(t) = _γ’_(t)_ p_(.) = _ c_(.)_	13	1494.6	196.7	0.000	0.000	32	6151.0
φ_ (.)_γ’’_ (.)≠_γ’_(t)_ p_(.) = _ c_(.)_	14	1501.2	203.3	0.000	0.000	32	6157.7
φ_ (.)_γ’’_(.) ≠_γ’_(.)_ p_(.) = _ c_(.)_	15	1508.8	210.9	0.000	0.000	25	6179.7
φ_ (.)_γ’’_(.) = _ γ’_(.)_ p_(.) = _ c_(.)_	16	1534.6	236.7	0.000	0.000	24	6207.6
φ_ (.)_γ’’0_ = _ γ’0 p_(.) = _ c_(.)_	17	1597.9	300.0	0.000	0.000	23	6272.9

Capture probability was allowed to vary with time between primary sampling periods and capture and recapture probability were assumed equal. Akaike information criterion corrected for small sample size (AICc) and was used to determine the best fitting model (in bold below). The notation ‘.’ indicates that a given parameter was kept constant and *t* indicates that a given parameter was allowed to vary with time.

φ Phi denotes survival.

p probability of capture.

c probability of recapture.

. denote constancy of the preceding parameter over time. Subscript t denotes that the parameter is time varying.

s denote time varying by austral season.

γ’’ = γ’ = 0  =  no emigration model.

γ’’ = γ’  =  random emigration model.

γ’’(t) ≠ γ’(t)  =  Markovian emigration model.

**Table 7:** Seasonal temporary emigration rates for the best fitting Markovian model using the overall dataset that included all juvenile and adult dolphins combined.

	Temporary emigration rates	
	(γ'')	(γ')
Season		
Autumn	0.29 (0.05)	0.52 (0.10)
Winter	0.00 (0.00)	0.34 (0.11)
Spring	0.14 (0.03)	0.35 (0.13)
Summer	0.06 (0.02)	0.97 (0.13)

There are two rates of temporary emigration: γ'' is the probability of being a temporary emigrant if the animal was present in the previous period while γ' is the probability of being a temporary emigrant if the animal was absent in the previous period. Standard error is shown in brackets.

## Discussion

### Abundance estimates are seasonally dependent

One of the most interesting results was the seasonal differences in abundance estimates of juveniles and adults combined, with consistently lower abundance estimated during winter months and higher during summer and autumn months. Specifically, abundance estimates varied from a low of 63 (95% CI 59-73) in winter 2007 to a high of 139 (95% CI 134-148) in autumn 2009.

Elsewhere, the abundance of coastal bottlenose dolphins in study areas of comparable size to this study (120 km^2^) have reported similar population estimates [1], [7], [9], [45], [46], [47], [48], [49]. For example, abundance of bottlenose dolphins in Port Stephens (140 km^2^), Jervis Bay (102 km^2^) and Clarence River (89 km^2^) ranged between 61 and 160 individuals [7], [9]. However, comparison of dolphin abundance estimates between studies should be treated with caution, particularly when sampling approaches vary. In contrast to our research, the studies noted above used closed population models which did not incorporate temporary emigration into their abundance estimates. However, these studies highlighted that individuals differed in their sighting frequency and classified individuals as resident, transient or occasional visitors [7], [9], which implies that individuals exhibited differing temporary emigration rates.

Perhaps more appropriate, is to compare population estimates of the bottlenose dolphin population in St Joseph Bay, Florida, U.S.A. (150 km^2^) to the Bunbury population given that both studies used the Robust Design to estimate seasonal abundances. In St Joseph Bay a resident community of 78–152 dolphins was documented. This population also exhibited a seasonal fluctuation in abundance with peak abundance detected in spring and autumn months resulting in estimates of 237–410 individuals during these periods. Although there was a difference in the timing, the seasonally-dependent fluctuation in abundance is similar between Bunbury and St Joseph Bay.

### Temporary emigration rates and apparent survival rates

This study highlights the importance of considering temporary emigration when estimating abundance of coastal bottlenose dolphins. In this study, Markovian models were better than both random temporary emigration models and models with no temporary emigration. Temporary emigration rates varied markedly between seasons for the best fitting Markovian model. The temporary emigration rates during time intervals when animals had been absent in the previous period were higher than the temporary emigration rates for those present in the previous period. This implies that some individual dolphins leave the study area for multiple seasons but subsequently return.

Other studies have also used the Robust Design to estimate temporary emigration rates of bottlenose dolphins [10], [38]. The rates of temporary emigration calculated for the Azores archipelago (∼5400 km^2^) were reportedly high between consecutive years 0.42 (± 0.12 SE) and 0.76 (± 0.05 SE) [10]. Similarly in the smaller coastal area of Shark Bay (226 km^2^), temporary emigration rates were relatively high: 0.33 (± 0.07 SE) – 0.66 (± 0.05 SE)) [38]. The temporary emigration rates estimated in these studies are similar to those for Bunbury (γ') 0.34–0.97 (± SE 0.11) which varied seasonally. Apparent survival rates were high and comparable between these three bottlenose dolphin studies: apparent adult survival rates for the Azores dolphins were slightly higher (0.97±0.02 SE) [10] than that that of juveniles and adults combined in Shark Bay (0.95±0.02 SE) and Bunbury (0.95±0.02 SE).

### Biological interpretation

The seasonally-dependent fluctuation in abundance is likely explained by the polygynous mating system typical of coastal bottlenose dolphins (*Tursiops* sp.). Long-term studies indicate that bottlenose dolphins live in mixed sex communities with looser bonds between adult females than adult males [50], [51] and, compared to males, females have smaller home ranges and stronger site fidelity [29], [30], [52]. For females, particularly mothers with calves, sheltered waters may be favoured over open coastal waters as they provide more protection from predation as well as more predictable prey resources [53]. In contrast, adult males range more widely, have lower site fidelity and larger home ranges, most likely as a result of optimising the number of encounters with adult females for mating opportunities [52] coupled with the dynamics of their social network which relies on bonds with fewer individuals than adult females [50]. The seasonally-dependent fluctuation in dolphin abundance observed in this study may be the result of an influx of adult males into the study area during the breeding season (summer/autumn season) and their subsequent departure during the non-breeding months. This is consistent with the documented peak calving and mating season in Bunbury spanning the summer and autumn months (unpublished data) and a twelve month gestation period.

The probability of being a temporary emigrant was estimated at zero during winter months when the population estimate was smallest. Furthermore, temporary emigration rates were higher during warmer seasons when males were in the study area for mating opportunities.These sex differences in ranging have been confirmed for bottlenose dolphins inhabiting other coastal waters in the USA [36], [49]. Therefore it may be expected in this study that some individuals, most likely males and non-reproductive females have home ranges much larger than the study area, resulting in temporary emigration when searching for prey and mating opportunities. In contrast, females, most likely mothers with calves, may have smaller home ranges and show more site fidelity by not emigrating and instead residing entirely within the study area year-round.

### Future research

Future research efforts should strive to document the sex of a larger proportion of the individuals in the study population than we achieved, as this information would allow for sex-specific estimation of abundance, survival probabilities and temporary emigration rates. The sampling regime used in this study was restricted by weather and could be improved by having shorter secondary sampling periods to ensure population closure. Specifically, the assumption of population closure within secondary periods could be optimised by completing these sampling periods in more closely-spaced time periods and with more temporally spaced primary periods, if weather conditions are favourable [11].

## Conclusions

The presented abundance, survival and temporary emigration estimates of the local dolphin population serve as an important baseline for future comparisons. As such, this research is the first step in science-informed management of the dolphins inhabiting the waters of the rapidly expanding city of Bunbury. Results highlight that mark-recapture models must accommodate the complexities of an animal’s life history and biology in order to produce meaningful and accurate abundance estimates. We have demonstrated that the Robust Design is suitable for estimating the population size and determining the apparent survival rates for coastal bottlenose dolphins that emigrate seasonally.
