# Correction: Use of the Robust Design to Estimate Seasonal Abundance and Demographic Parameters of a Coastal Bottlenose Dolphin (*Tursiops aduncus*) Population

**DOI:** 10.1371/annotation/369119db-d9ca-4473-9390-89ee0c2a532f

**Published:** 2013-10-24

**Authors:** Holly C. Smith, Ken Pollock, Kelly Waples, Stuart Bradley, Lars Bejder

This article was republished on October 23, 2013 due to an incorrect figure. Please download this article again to view the figure. 

